# Investigation of the Structure and Functional Activity of the YqeK Protein in *Streptococcus pyogenes* with High Efficiency in Hydrolyzing Ap4A

**DOI:** 10.3390/microorganisms13020230

**Published:** 2025-01-22

**Authors:** Kai Yang, Suhua Hu, Yao Yao, Kaijie Li, Zunbao Wang, Xinyu Wang, Dan Ma, Mingfang Bi, Xiaobing Mo

**Affiliations:** State Key Laboratory for Diagnosis and Treatment of Severe Zoonotic Infectious Diseases, Key Laboratory for Zoonosis Research of the Ministry of Education, Institute of Zoonosis, College of Veterinary Medicine, Jilin University, Changchun 130062, China; yangkai99101@163.com (K.Y.); 15149001998@163.com (S.H.); 15843189107@163.com (Y.Y.); 17838408328@163.com (K.L.); wangzunbao@163.com (Z.W.); wxy0209912@163.com (X.W.); madandan@jlu.edu.cn (D.M.)

**Keywords:** antimicrobial resistance, antimicrobial, *Streptococcus pyogenes*, *Sp-yqeK*, enzymatic activity, Ap4A, crystal structure

## Abstract

*Streptococcus pyogenes* is an important zoonotic Gram-positive bacterium that appears in chains, without spores or flagella, and belongs to the *beta-hemolytic streptococci*. It can be transmitted through droplets or contact, with the preferred antibiotics being penicillin, erythromycin, or cephalosporins. However, the misuse of these drugs has led to antibiotic resistance, posing a significant threat to both human and animal health. Studying resistance genes encoding proteins is crucial for mitigating the emergence of resistant strains and improving treatment outcomes. Interestingly, a dinucleotide known as diadenosine tetraphosphate (Ap4A) exists in *Streptococcus pyogenes*; its accumulation in response to various stress signals can inhibit bacterial pathogenicity and enhance antibiotic susceptibility. Our research focuses on the *Sp-yqeK* protein, which we have identified as a hydrolase that symmetrically cleaves Ap4A. The *Sp-yqeK* protein effectively cleaves Ap4A, producing adenosine diphosphate (ADP) molecules. Results indicate that this enzyme exhibits optimal activity at pH 7.0 and a temperature of 45 °C. Furthermore, we determined the crystal structure of the *Sp-yqeK*, Mg^2+^, and ADP complex at a resolution of 2.0 Å, providing insights into the interactions crucial for catalytic efficiency between *Sp-yqeK* and ADP. This complex reveals unique folding characteristics of the HD domain superfamily proteins, accommodating both ADP and Mg^2+^. These components are securely embedded into the polar cavity of the yqeK protein through conserved residues (His^29^, Lys^62^, His^91^, His^117^, Asp^135^, Leu^172^, Phe^180^, and Thr^183^), highlighting the residues responsible for Ap4A hydrolysis and Mg^2+^ binding. Our research offers a deeper understanding of the hydrolysis mechanism of Ap4A and the specificity of *Sp-yqeK*, providing structural insights that may support future studies on antibiotic resistance in *Streptococcus pyogenes* and other Gram-positive bacteria.

## 1. Introduction

*Streptococcus pyogenes* is a significant Gram-positive bacterium with a strong infectious potential, primarily causing respiratory infections that can lead to tissue necrosis and even death in severe cases [1,2]. The treatment of *Streptococcus pyogenes* primarily relies on antibiotics, such as penicillin and azithromycin. However, with prolonged use and inappropriate utilization of these medications, the bacterium has developed resistance to certain antibiotics [3,4].

*Streptococcus pyogenes* can produce various adenosine polyphosphate nucleotides (APnNs) to adapt to the host environment. Among them, the most thoroughly studied is Ap4A, which is a tetranucleotide composed of two adenosine nucleotides linked by four phosphates at the 5′-5′ position. It is widely present in both prokaryotes and eukaryotes [5,6,7]. Ap4A is produced during the aminoacylation reaction, when aminoacyl-tRNA synthetase catalyzes the incorporation of amino acids into proteins. GlyRS is a tRNA synthetase that directly mediates the synthesis of Ap4A [8]. Under conditions of increased external stimulation, Ap4A rapidly accumulates within bacteria, altering their survival ability and physiological functions [9,10]. The accumulation of Ap4A affects bacterial physiological functions in multiple ways. Elevated levels of Ap4A disrupt the timing of cell division, reduce metabolic capabilities, and increase sensitivity to aminoglycoside antibiotics, heat, and oxidative stress [6,11,12]. Compared to ATP, Ap4A plays a more significant role in regulating gene expression, and it can induce apoptosis during cellular stress [13]. Moreover, when *Escherichia coli* is disrupted by kanamycin, the bacteria produce a stress response, leading to a significant increase in Ap4A concentration. Ap4A can enhance *E. coli*’s sensitivity to kanamycin by inhibiting biofilm formation and the quorum sensing (QS) pathway. Furthermore, it can restrict bacterial motility [14]. When pathogenic *pseudomonas aeruginosa* experiences stress under conditions such as high temperature and oxidation, it increases the concentration of Ap4A. This leads to a reduction in bacterial resistance to aminoglycoside antibiotics, thereby enhancing the effectiveness of these antibiotics [15]. Additionally, Ap4A can potentially promote the assembly of a cGAS-independent stimulator of interferon genes (STING) signaling complex in the nucleus by binding to STING. This suggests that Ap4A may play an important role in activating immune responses [16,17,18].

Hydrolases or phosphatases have evolved to maintain cellular homeostasis of Ap4A. The two main families of Ap4A hydrolases are the Nudix and ApaH families [19,20]. It is worth noting that the hydrolases of the ApaH family catalyze the symmetrical cleavage of Ap4A, producing two ADP molecules [21,22,23].Meanwhile, the Nudix family of Ap4A hydrolases cleaves Ap4A asymmetrically [24,25,26], generating adenosine triphosphate (ATP) and adenosine monophosphate (AMP), which are commonly found in eukaryotes and bacteria [27,28,29]. Therefore, the activity of ApaH and Nudix hydrolases is crucial for pathogenic bacteria when invading host cells. Additionally, Ap4A phosphohydrolase converts Ap4A into ADP and ATP in the presence of phosphate. The absence of this process can lead to a sustained stress response, hindering bacterial growth and proliferation. In recent years, yqeK from *Staphylococcus aureus* has been identified as a hydrolase capable of symmetrically cleaving Ap4A. YqeK is widely distributed in Gram-positive bacteria and is largely conserved [30,31]. Among the known NpnN hydrolases, yqeK is most similar to ApaH, which is the primary Ap4A hydrolase in Proteobacteria.

YqeK exhibits specific specificity for Ap4A, symmetrically cleaving it into ADP molecules. The catalytic efficiency of yqeK is at least twice as high as other tested diadenosine tetraphosphates. Knocking out the *yqeK* gene in mutant *Streptococcus* resulted in elevated Ap4A levels. Compared to the wild type, the mutants showed early growth delay, inhibited biofilm formation, and reduced production of water-insoluble extracellular polysaccharides [32]. By inhibiting yqeK activity, elevated levels of Ap4A can enhance the effectiveness of antibiotic-induced bacterial cell death. Previously, the crystal structures of yqeK from *Halobacillus halophilus* (*Ha*-*yqeK*), *Streptococcus agalactiae* (*Sa-yqeK*), and *Clostridium acetobutylicum* (*Ca*-*yqeK*) were determined using crystallography methods at different resolutions (PDB structures, from the Joint Center for Structural Genomics, unpublished). Although the alignment of yqeK suggests a metal-dependent cleavage mechanism between the β- and γ-phosphates of the substrate, the molecular mechanism of hydrolysis has not been elucidated. The previous studies explored the role of Fe^3+^ in yqeK (SP1746) [33]. However, the active sites of divalent metal ions and related research have not yet been explained. Therefore, further understanding of the structure and function of the yqeK hydrolase in *Streptococcus pyogenes* is crucial.

This study examined the Ap4A hydrolase activity of yqeK under different pH and temperature conditions, with an optimal pH of ~7.0 and an ideal temperature of ~45 °C. Additionally, the crystal structure of the yqeK, Mg^2+^, and ADP complex was determined at a resolution of 2.07 Å, revealing the key active site residues involved in the symmetrical hydrolysis of Ap4A into ADP molecules by yqeK. Our research provides an understanding of the Ap4A hydrolysis mechanism and the specific structure of yqeK, offering structural insights that could aid future studies on antibiotic resistance.

## 2. Materials and Methods

### 2.1. Cloning, Expression, and Purification of Streptococcus pyogenes yqeK Protein or Mutant Proteins

The nucleotide sequence encoding the yqeK protein (GenBank ID: 8WMY_A) was retrieved from the NCBI database. The pET28b plasmid (Sangon, Shanghai) was linearized using the restriction enzymes *Nde* I and *Xho* I, and the target fragment was ligated into the pET28b expression vector. After successful verification by double enzyme digestion, PCR, and sequencing, the recombinant plasmid containing the *yqeK* sequence was transformed into *Escherichia coli* BL21 (DE3) for protein expression. The transformed cells were cultured in Luria-Bertani (LB) medium containing 1 mM kanamycin, shaking at 220 rpm at 37 °C. When the culture reached an OD_600_ of 0.6, protein expression was induced by adding 0.4 mM isopropyl β-D-1-thiogalactopyranoside (IPTG). The bacteria were induced at 20 °C and 220 rpm for 12 h. After induction, bacteria were collected by centrifugation at 4000× *g* for 15 min. The resulting cell pellet was resuspended in lysis buffer containing 20 mM Tris-HCl (pH 7.9), 25 mM potassium phosphate (pH 6.8), 500 mM NaCl, 10% glycerol, 100 nM PMSF, and 1 mM DTT. The resuspended cells were lysed using a high-pressure homogenizer (JNBIO, Guangzhou, China) at 4 °C, with four passes to ensure thorough disruption. The lysate was centrifuged at 4 °C and 12,000× *g* for 1 h to obtain the supernatant.

Protein purification was initially performed using Ni-NTA affinity chromatography. First, recharge the purification column with Ni²⁺ using 250 mM NiCl₂, followed by sample loading at a flow rate of 1 mL/min. Elution was carried out using an imidazole gradient. The eluted protein was dialyzed overnight in a low-salt buffer (20 mM Tris-HCl, 200 mM NaCl). Next, further purification was conducted using a HiTrap Heparin HP column (Cytiva, Marlborough, MA, USA). The column was equilibrated with a balance buffer (25 mM Tris-HCl, 0.1 mM EDTA, 0.4 mM DTT), and linear elution was performed using an elution buffer (25 mM Tris-HCl, 0.1 mM EDTA, 0.4 mM DTT, 1 M NaCl). Finally, gel filtration chromatography was performed using a HiLoad Superdex-200 26/60 column (Cytiva, Marlborough, MA, USA) with a gel filtration buffer containing 25 mM Tris (pH 7.5), 500 mM NaCl, 5 mM EDTA, and 4 mM DTT. The entire purification process was carried out using a ClearFirst-3000 Purifier protein purification system (Flash, Beijing, China).

Mutant proteins were obtained through site-directed mutagenesis. Based on the structure of *Sp-yqeK*, several potential mutation sites were identified, and target mutagenesis primers were designed. The primers included the desired mutated amino acid sequences, and plasmids were obtained by PCR amplification. The PCR products were ligated into pET28b using restriction endonucleases *Nde* I and *Xho* I. Subsequent purification methods were consistent with those used for the wild-type yqeK protein.

The purified yqeK protein was dialyzed into a storage buffer containing 20 mM Tris-HCl (pH 7.5), 10 mM MgCl_2_, and 500 mM NaCl. To enhance storage stability, 100 nM PMSF and 35% glycerol were added. The protein was aliquoted and stored at −80 °C for future use.

### 2.2. Crystallization, Data Collection and Structure Determination

A portion of the partially purified protein was centrifuged multiple times at 5000× *g* at 4 °C and concentrated through filtration, achieving a final concentration of ~10 mg/mL. The concentrated *Sp-yqeK* protein was subjected to crystallization tests using the hanging drop vapor diffusion method in a 24-well plate [34]. Over 500 conditions were screened using a crystal screening kit from Hampton Research. Under conditions of 20 °C, 1.0 μL of protein was mixed with 1.0 μL of reservoir solution containing 12% isopropanol, 18.5% PEG-4000, and 100 mM Hepes sodium (pH 7.4), which was balanced over 1 mL of reservoir solution (optimized crystallization buffer), resulting in the formation of the best crystals. Following optimization, we flash-froze single crystals using a cryo-solution, which included the crystallizing buffer plus 30% glycerol. Data were collected at the Taiwan synchrotron radiation center and were processed using the HKL2000 (v712) software. Utilizing 2OGI as the search model, we solved the crystal structure with MORDA/CCP4 and visualized it using the Coot (v8.0.012) software (www.ccp4.ac.uk). The data processing, as well as refinement statistic details of the structure, are listed in Table 1.

### 2.3. Hydrolysis Activity Assay of Sp-yqeK on Ap4A

The hydrolysis activity assay of *Sp-yqeK* on Ap4A is accomplished by measuring the levels of ADP, which is a product of ATP dephosphorylation and can be rephosphorylated back to ATP. These dephosphorylation and phosphorylation processes are mediated by various phosphatases, phosphorylases, and kinases, with the conversion of ADP to ATP primarily occurring in mitochondria and chloroplasts. Traditionally, ADP levels are determined using luciferase or luciferin-based assays. However, the luciferase system is unstable, and luminescence detection equipment is not commonly found in many laboratories. To address this issue, the ADP assay kit from Abnova (colorimetric/fluorescent method, Catalog No.: KA0807) is used to measure ADP levels via fluorescence. In this experiment, ADP is converted into ATP and pyruvate. The resulting pyruvate is quantified by fluorescence (Ex: 535 nm; Em: 587 nm). This method is simple, sensitive, stable, high-throughput, and versatile, capable of detecting ADP concentrations as low as 1 μM in biological samples. According to this method, 1~50 μL of samples are added to each well of a 96-well plate and adjusted to 50 μL with the assay buffer (formulation). Then, 50 μL of the reaction mixture is added to each well. Reactions are performed with various concentrations of ADP (0~1.0 nmol), and fluorescence values are read to construct a standard curve with ADP concentration on the X-axis and Relative Fluorescence Units (RFU) on the Y-axis. After incubating in the dark at room temperature for 30 min, the ADP concentrations are calculated based on the ADP standard curve. Parallel treatments of ADP and Ap4A standards are conducted in the absence of *Sp-yqeK*. To determine potential hydrolysis, we compared the retention times of the sample peaks with those of ADP and Ap4A standards. The samples are analyzed using high-performance liquid chromatography (HPLC), and the resulting peak profiles are compared.

### 2.4. Investigation of the Optimal Temperature for the Hydrolytic Activity of Sp-yqeK on Ap4A

Incubate in the dark at temperatures of 15, 25, 35, 45, 55, and 65 °C for 30 min. Subsequently, quantify the ADP content in the incubation products using the ADP Assay kit, and determine the *Sp*-*yqeK* enzyme activity based on this standard.

### 2.5. Investigation of the Optimal pH for the Hydrolytic Activity of Sp-yqeK on Ap4A

Test the activity of *Sp-yqeK* at different pH values (100 mM Acetic acid-sodium acetate (pH 5~6), Tris-HCl (pH 7~9), Carbonate (pH 9~11) and Boric acid-sodium borate (pH 12) buffer) solutions with pH values ranging from 5.0 to 12.0. Use a pH meter to adjust the pH of the different buffer solutions. The incubation temperature for the entire catalytic reaction is set at 45 °C.

### 2.6. Research on the Optimal Divalent Metal Ions for the Hydrolytic Activity of Sp-yqeK on Ap4A

To investigate the most dependent divalent metal ions, incubate with EDTA, Zn^2+^, Mg^2+^, Ca^2+^, Mn^2+^, and Ni^2+^ in the dark for 30 min, and then measure the activity.

### 2.7. Investigation of the Hydrolytic Efficiency of Sp-yqeK on Different Concentrations of Ap4A Under Optimal Conditions

We measured the amount of ADP produced by the wild-type and different mutant *Sp-yqeK* (2 µM) in response to 1 mM substrate Ap4A within 30 min. The Km value provides the most intuitive measure of enzyme catalytic efficiency, known as the specificity constant. It not only reflects the enzyme’s affinity for the substrate and its catalytic ability but can also be used to compare the catalytic efficiency of different enzymes on a specific substrate. To determine this, WT and various active mutant *Sp-yqeK* protein concentrations (0.1 mg/mL) were added, along with Ap4A concentrations ranging from 0.1 to 4 × 10^3^ μM, to generate enzyme kinetics curves. The different Ap4A substrate concentrations were plotted on the X-axis and the reaction rates on the Y-axis. Linear fitting was performed using the Lineweaver-Burk double-reciprocal method to plot the kinetics curve of *Sp-yqeK* and calculate the kinetic constant Km.

### 2.8. Structural Determination of the Sp-yqeK-Ap4A Complex from Streptococcus pyogenes

Crystals that had previously grown well were rapidly frozen in a storage solution containing 20% glycerol. Single-wavelength diffraction data were collected at the Taiwan Synchrotron Radiation Resource Center (NSRRC) and processed using HKL2000 (v712) software [35]. The structure was determined using Molecular Replacement/Phenix (https://phenix-online.org/) with 2OGI as the search model. The model was built using the Coot program and refined to a resolution of 1.91 Å using refine/Phenix. The crystallographic statistics for this structure are listed in Table 1. Structural and surface diagrams were generated using Chimera (v1.15) software (www.cgl.ucsf.edu/chimera). To confirm whether the residues involved in Mg^2+^ coordination are the catalytic residues required for Ap4A hydrolysis, alanine mutations were introduced at these key residues, and hydrolysis experiments were conducted. The collected data were processed, and an initial model was generated using Phenix (v1.21.2) software (https://phenix-online.org/), along with the calculation of the Fo-Fc Fourier difference map. A visual model of the *Sp-yqeK*-Ap4A complex was constructed by overlaying the characteristic sugar backbone and adenine of ADP/Ap4A. 

## 3. Results

### 3.1. Investigation of the Functional Activity of Sp-yqeK

**Figure 1:**
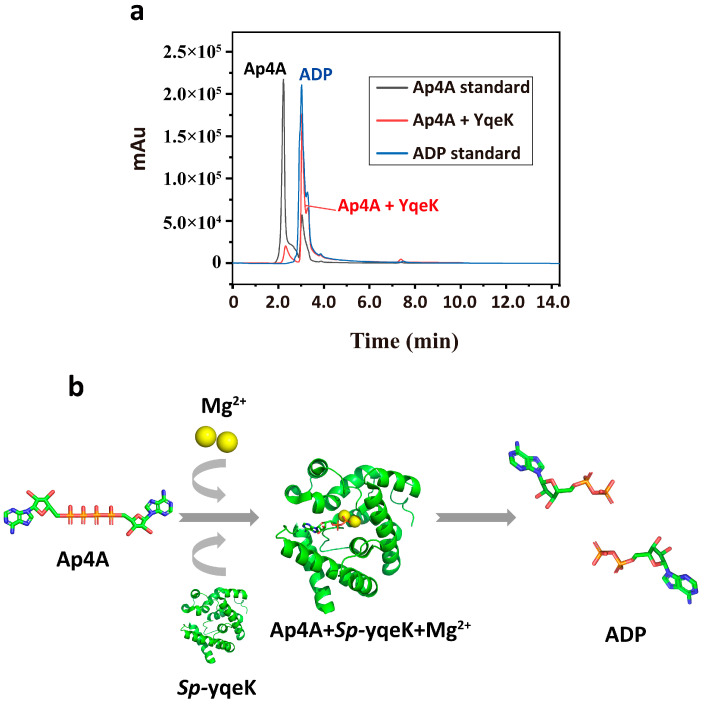
Investigating the function of *Sp-yqeK*. (**a**) The HPLC chromatogram shows the peak profiles of the reaction between *Sp-yqeK* and Ap4A (254 nm), as well as the standard peaks for ADP and Ap4A. The black, blue, and red lines represent the peaks for Ap4A, ADP standard, and the reaction between *Sp-yqeK* and Ap4A, respectively, confirming the conversion of Ap4A to ADP. (**b**) Diagram of the reaction in the presence of Mg^2+^ where *Sp-yqeK* hydrolyzes an Ap4A molecule into two ADP molecules.

According to SDS-PAGE analysis, the molecular weight of *Sp-yqeK* is approximately 23 kDa, which is consistent with the expected size (Supplementary Figure S1). To assess whether *Sp-yqeK* can hydrolyze Ap4A, we incubated *Sp-yqeK* with Ap4A for 20 min. Following the incubation, the samples were analyzed using positive-phase high-performance liquid chromatography (HPLC). The results showed that the retention time of the sample peak shifted from that of the Ap4A peak towards the ADP peak, indicating that *Sp-yqeK* has the ability to hydrolyze Ap4A into ADP. The peak corresponding to the retention time of the ADP standard was identical to that of the sample, with the peak shapes also matching. Furthermore, all hydrolyzed Ap4A was detected at the Ap4A standard peak. By comparing the shapes and retention times of the peaks, we conclude that *Sp-yqeK* effectively decomposes Ap4A into ADP molecules (Figure 1).

### 3.2. Investigation of the Optimal Hydrolytic Activity of Sp-yqeK

**Figure 2:**
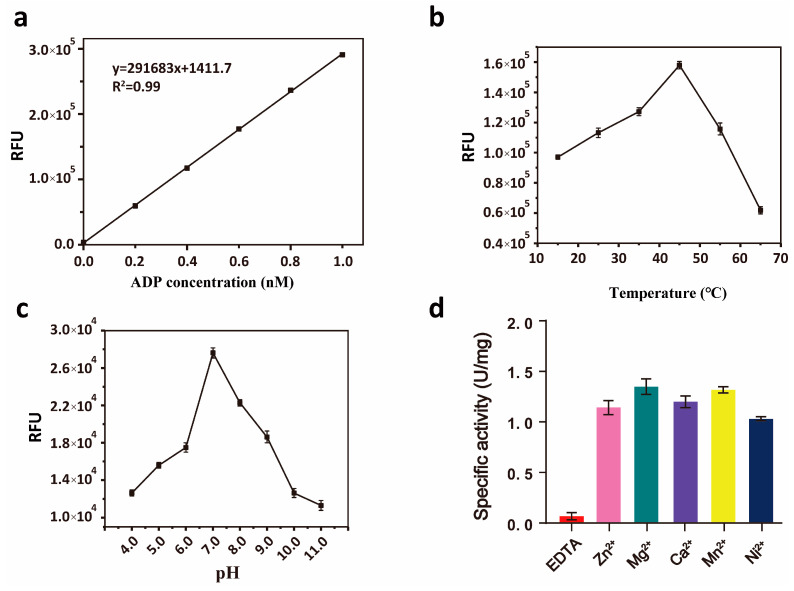
Exploration of the optimal conditions for *Sp-yqeK* activity. (**a**) A standard curve for ADP production was established to measure subsequent enzyme activity. (**b**,**c**) The curves of *Sp-yqeK* activity in response to changes in temperature and pH. (**d**) The effect of divalent metal ions on the activity of *Sp-yqeK*.

**Figure 3:**
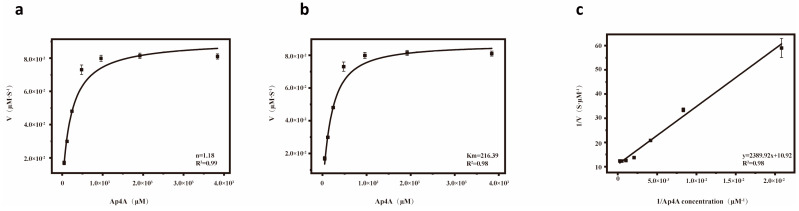
*Sp-yqeK* enzyme kinetics curve. (**a**) Hill kinetics curve of *Sp-yqeK*, with the concentration of Ap4A on the x-axis and the rate on the y-axis. (**b**) Michaelis–Menten kinetics curve of *Sp-yqeK*, with the concentration of Ap4A on the x-axis and the rate on the y-axis. (**c**) Lineweaver–Burk double-reciprocal plot of *Sp-yqeK*, with the reciprocal concentration of Ap4A on the x-axis and the reciprocal rate on the y-axis.

Hydrolases exhibit optimal activity under specific conditions. First, a standard curve of ADP fluorescence values at various concentrations was established (Figure 2a). The optimal reaction temperature and pH for *Sp-yqeK* were then determined. Similar to most enzymes, *Sp-yqeK* exhibits maximum activity at specific temperature and pH levels. The optimal reaction temperature for *Sp-yqeK* was found to be approximately 45 °C (Figure 2b). The enzyme’s catalytic activity increased within the pH range of 5.0 to 7.0 but declined and became inactive in the pH range of 8.0 to 12.0. Therefore, the optimal pH for *Sp-yqeK* was determined to be around 7.0 (Figure 2c). Additionally, *Sp-yqeK* is a metal ion-dependent hydrolase (Table 2 and Figure 2d), showing varying degrees of relative enzyme activity depending on the metal ion present, with higher activity observed for Mg^2+^ and Mn^2+^. The inclusion of EDTA significantly reduced the relative enzyme activity, indicating that *Sp-yqeK* requires metal ions for its activity, and that EDTA chelation of these ions impairs its function. A plot of substrate concentration versus initial reaction rate showed that the enzyme-catalyzed reaction rate increases with higher substrate concentrations (Figure 3a). For WT-*Sp-yqeK*, the reaction rate plateaued at a substrate concentration of 2 × 10^3^ μM, suggesting full binding of *Sp-yqeK* to the substrate Ap4A at this concentration (Figure 3b). Furthermore, a Lineweaver-Burk double-reciprocal plot was constructed to determine the Km value for *Sp-yqeK* (Figure 3c). WT-*Sp-yqeK* exhibited the lowest Km value, ~216 µM.

### 3.3. Crystal Structure of the Sp-yqeK-Mg²⁺-ADP Complex

To elucidate the molecular mechanism of *Sp-yqeK*-mediated Ap4A cleavage and the structural role of di-ions in its catalytic mechanism, we conducted crystallization screening and structural determination of *Sp-yqeK* complexes with di-ions and/or Ap4A. Among the screened ions, *Sp-yqeK* successfully crystallized in the presence of Mg^2+^ and Ap4A. The *Sp-yqeK*-Mg^2+^-ADP complex crystallized in the C121 space group, with unit cell dimensions of a = 159.944 Å, b = 37.672 Å, c = 76.301 Å, and β = 92.704°. Using 2OGI as a search model, the structure of *Sp-yqeK* in complex with Mg^2+^ and ADP was determined via molecular replacement (MR) in Phenix, followed by model refinement and rebuilding using Refine/Phenix and Coot. The asymmetric unit contains two *Sp-yqeK*-Mg^2+^-ADP complexes, and crystallographic statistics are summarized in Table 1.

**Figure 4:**
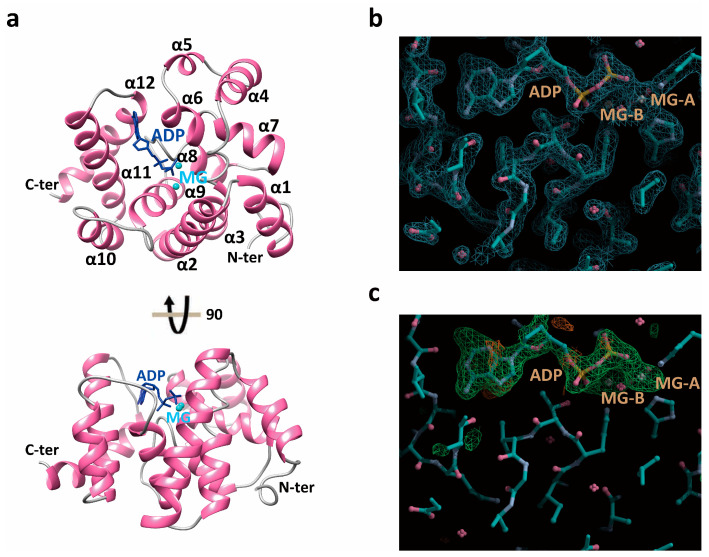
Structure of *Sp-yqeK* protein. (**a**) The upper and lower images show different perspectives of the crystal structure of the *Sp-yqeK*, Mg^2+^, and ADP complex, highlighting the 12 alpha helices, as well as the N-terminal and C-terminal regions. (**b**) Displays the omit map of ADP and Mg^2+^. (**c**) Shows the 2mFo-DFc electron density map.

**Figure 5:**
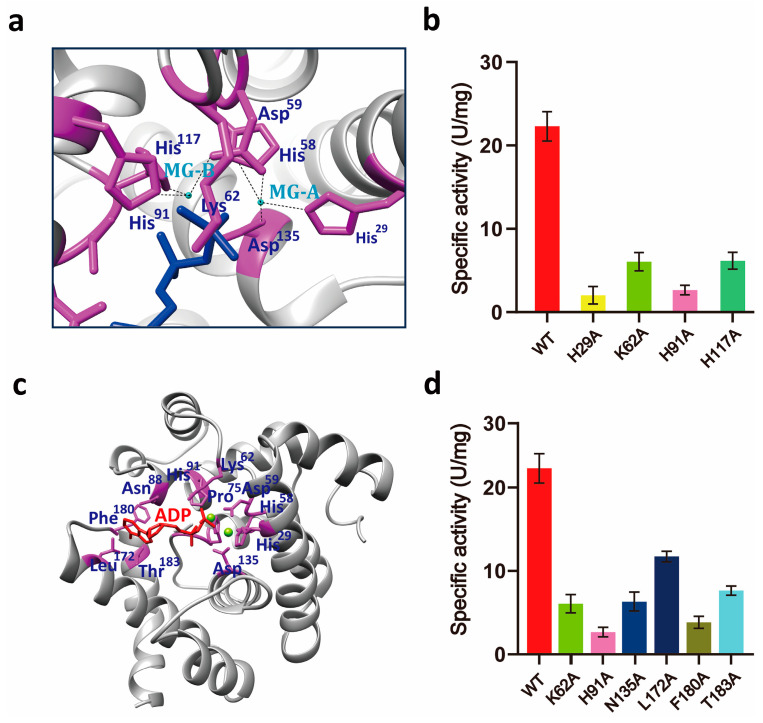
Structural details of the *Sp-yqeK*, Mg^2+^, and ADP complex. (**a**) Close-up view of the Mg^2+^-binding residues in the active site, indicated by purple labels. (**b**) Catalytic activity of *Sp-yqeK* mutant proteins with single mutations in residues involved in di-ion binding. (**c**) Close-up view of ADP-binding residues in the active site. The side chains of these residues are labeled in purple, while ADP is labeled in deep blue. (**d**) Catalytic activity of *Sp-yqeK* mutant proteins with single mutations in residues involved in ADP binding.

**Figure 6:**
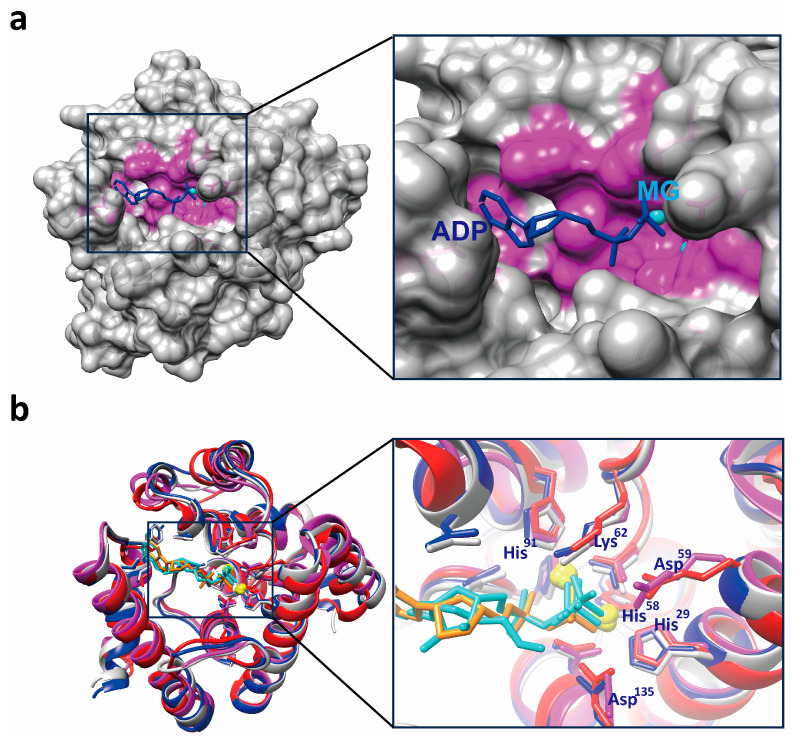
Active site of Ap4A hydrolysis in *Sp-yqeK*. (**a**) YqeK structure depicting the ADP molecule in the binding pocket. (**b**) Left panel: Structural comparison of yqeK from different species. Crystal structure of *Halalkalibacterium halodurans* yqeK complexed with FE and ADP-like ligand (PDB: 2O08). YqeK, FE, and ADP-like ligand are shown in red, green, and yellow, respectively. Crystal structure of *Streptococcus agalactiae* yqeK complexed with GDP and FE (PDB: 2OGI). YqeK, FE, and GDP are shown in deep blue, green, and yellow, respectively. Crystal structure of *Clostridium acetobutylicum* yqeK complexed with GDP and PO4 (PDB: 3CCG). YqeK, FE, and PO4 are shown in purple, green, and yellow, respectively. Crystal structure of *Strephylococcus pyogenes* yqeK complexed with Mg^2+^ and ADP (PDB: 8WMY). YqeK, Mg^2+^, and ADP are shown in gray, brown, and yellow, respectively. Right panel: Conserved residues involved in di-ion binding.

The Fo-Fc Fourier difference map revealed strong spherical peaks at the dimer interface, identified as magnesium ions Mg^2+^-A and Mg^2+^-B (Figure 4b,c, and Supplementary Table S1). Structural analysis identified key interacting residues: His29, His^58^, Asp^59^, and Asp^135^ coordinating Mg^2+^-A, and Asp^59^, His^91^, and His^117^ coordinating Mg^2+^-B (Figure 5a). Alanine mutations at His^29^, Lys^62^, His^91^, and His^117^ significantly reduced Ap4A cleavage activity (Figure 5a,b, and Table 3 and Table 4). Additionally, the mutation of Asp^59^, which coordinates both Mg^2+^-A and Mg^2+^-B, resulted in protein aggregation, emphasizing its critical role in structural integrity and catalytic function. Within the *Sp-yqeK*-Mg^2+^-ADP complex, ADP was tightly positioned in the active site (Figure 5c,d). The interactions involved extensive hydrogen bonding and hydrophobic contacts, highlighting *Sp-yqeK*’s role in substrate recognition and binding. Key residues, including His^29^, Lys^62^, His^91^, His^117^, Asp^135^, Leu^172^, Phe^180^, and Thr^183^, were identified as critical for these interactions. Based on the Michaelis–Menten kinetics equation, *Sp-yqeK* exhibited a low Km value (~216 μM), indicating high substrate affinity (Table 4). The substrate-binding pocket of *Sp-yqeK* was observed to be rectangular, measuring ~9 Å × ~5 Å × ~6.5 Å (width × length × depth) (Figure 6a). As expected, Ap4A was hydrolyzed into ADP. Conserved residues, including His^29^, Lys^62^, His^91^, His^117^, Asp^135^, Leu^172^, Phe^180^, and Thr^183^, anchored ADP binding alongside the two magnesium ions (Figure 4c). The bound ADP adopted an extended conformation, with the phosphate group projecting outward and the adenine moiety fitting inward into the pocket (Figure 4a). Multiple sequence alignments revealed conservation of most ADP/GDP-binding residues (Supplementary Figure S2). Structural comparisons showed ~40% sequence identity with previous structures, yet the positions of conserved side chains were highly similar (Figure 4b and Supplementary Figure S3). These findings suggest that *Sp-yqeK* shares a conserved substrate-binding mode and catalytic mechanism.

After aligning adenosine diphosphate to fit Ap4A into the complex structure, the visualization model revealed that one Ap4A molecule is enclosed within the active site of *Sp-yqeK*. The adenine moiety of the second ADP fragment slightly protrudes outward, while the catalytic site is positioned near Mg^2+^-A (Supplementary Figure S4). Based on this observation, we propose that *Sp-yqeK* binds Ap4A within its binding pocket, facilitates its hydrolysis, and subsequently releases the resulting ADP fragments.

## 4. Discussion

*Streptococcus pyogenes* is an important pathogen capable of causing multiple infections in humans or animals, and even death [2]. In recent years, *Streptococcus pyogenes* has developed varying degrees of resistance to drugs used to inhibit it [3,4]. There is a protein in *Streptococcus pyogenes* that can hydrolyze Ap4A [30].

Ap4A is a dinucleotide consisting of four phosphate groups that exist in organisms and regulates mRNA stability and gene expression in bacteria. Enzymatic reactions indicate that yqeK can effectively hydrolyze Ap4A in bacteria, producing two ADP molecules. Among the known Ap4A hydrolases, yqeK is similar to the major hydrolase ApaH found in *Aspergillus*. However, interestingly, while yqeK and ApaH share functional similarities, they belong to different families: the serine/threonine phosphatase family and the HD domain superfamily, respectively. Phylogenetic analysis shows that yqeK and ApaH appear in a mutually exclusive manner among bacterial species, with yqeK present in all species that lack ApaH [34,35,36,37,38]. Therefore, it can be established that yqeK can serve as a breakthrough target for reducing the pathogenicity of Gram-positive bacteria.

In our study, we identified the yqeK protein in *S. pyogenes* as an enzyme that symmetrically cleaves Ap4A and determined its enzymatic activity. Our analysis indicates that *Sp-yqeK* can effectively hydrolyze Ap4A in bacteria, resulting in the production of ADP molecules. The purified *Sp-yqeK* was used for enzymatic kinetic studies, and the optimal conditions for *Sp-yqeK*-catalyzed Ap4A hydrolysis were optimized. The most suitable reaction environment for *Sp*-*yqeK* is at pH 7.0. Changes in pH can affect enzyme activity by causing protein denaturation under extreme acidic or alkaline conditions, as well as influencing the dissociation between the enzyme’s active center, related groups, and the substrate. *Sp-yqeK* exhibits the highest activity at 45 °C. Temperature changes can affect enzyme activity in two ways: it can accelerate the reaction, as with ordinary chemical reactions, but higher temperatures can also lead to loss of activity due to the yqeK’s composition. The most optimal divalent metal ion for *Sp-yqeK* is Mg^2+^, which, compared to the previously analyzed Fe^3+^, exhibits much greater stability at the optimal pH, is more soluble in water, and has lower cytotoxicity.

To reveal the structural details of *Sp-yqeK* binding with optimal divalent metal ion, we tried to remove the possible binding metal ion during multiple protein purification steps and add optimal amount of MgCl_2_ to prepare the complex of yqeK, ADP and Mg^2+^ for crystallization. Moreover, from the structural analysis, the crystal structure of *Sp-yqeK* reveals a unique fold that accommodates ADP molecules and Mg^2+^. This finding emphasizes the potential of the divalent metal magnesium ion in stabilizing the transition state of the catalytic reaction or assisting in the binding of Ap4A to *Sp-yqeK*.

The structures of *Sa*-yqeK and *Sp-yqeK* (our structure) were determined in complex with GDP and ADP, respectively. A comparative analysis of the *Sp-yqeK*-ADP complex and the *Sa*-*yqeK*-GDP complex clearly demonstrates that ADP and GDP occupy similar positions within the active site of *Sp-yqeK*. ADP and the ions are nestled in a polar pocket formed by conserved residues of *Sp-yqeK* (His^29^, Lys^62^, His^91^, His^117^, Asp^135^, Leu^172^, Phe^180^, and Thr^183^), revealing the amino acids responsible for Ap4A hydrolysis. These amino acid residues play an important role in the structure and function of *Sp-yqeK* and may serve as targets for the development of novel drug inhibitors against *Streptococcus pyogenes* and other Gram-positive bacteria in the future. Additionally, we identified the catalytic active site of *Sp-yqeK* for Ap4A hydrolysis, which can be screened for small molecular drugs that effectively reduce *Sp-yqeK* activity and assess their functional characteristics [39]. We conducted a Protein-BLAST analysis of the amino acid sequence of *Sp-yqeK* and found similarities only with yqeK proteins from different bacteria. For host cells, no potential targets have been identified in the database so far, which may reduce the risk of off-target effects. Nevertheless, the possibility of off-target effects still exists, such as impacts on host metabolic pathways, immune system, gut microbiota, etc., which requires comprehensive evaluation. Ap4A is produced in large quantities when *Streptococcus pyogenes* invades, and it can reduce biofilm formation and slow the growth of the bacteria [32]. *Sp-yqeK* hydrolyzes Ap4A, significantly reducing its levels, thereby mitigating the threat of Ap4A. Developing small molecule drugs targeting the mutation sites of *Sp-yqeK* could inhibit Streptococcus pyogenes. However, most antibacterial or bacteriostatic molecules tend to develop resistance, such as mutations in active sites, bypassing the yqeK hydrolysis pathway of Ap4A through other metabolic pathways, or removing future small molecule drugs via active efflux pumps. These are important considerations for future design and research of small molecule drugs targeting the *Sp*-*yqeK* protein. Although this study has yielded some important findings, there are certain limitations that need to be considered due to the scope of the study and resource constraints. For example, the range of analysis on mutation sites is insufficient, lacking investigation into the impact of mutated yqeK protein on biofilm formation by *Streptococcus pyogenes* and its effect on antibiotic sensitivity. Additionally, further in-depth studies, such as in vivo experiments, are needed to explore potential off-target effects.

In summary, this holds promise as a new strategy for weakening the response of Gram-positive bacteria to stress signals and reducing their pathogenicity. In the future, we will further validate the potential role of this protein from the perspectives of in vivo experiments in *Streptococcus pyogenes* and small molecular drugs.

## Figures and Tables

**Table 1:** Data collection and refinement statistics.

Data Collection	*Sp-yqeK* in Complex with ADP and Mg
Space group	C 1 2 1
PDB ID	8WMY
Wavelength (Å)	1.0000
Cell dimensions	
a (Å)	159.944
b (Å)	37.672
c (Å)	76.301
α, β, γ (°)	90, 92.704, 90
Molecule/ASU	dimer
Resolution range (Å) ^a^	28.25–1.91 (1.978–1.91)
Rsym (%) ^a^	7.6 (40.2)
I/(I)	44.1 (6.3)
Completeness (%) ^a^	98.40 (98.40)
Redundancy ^a^	11.0 (10.9)
**Refinement**	
Search Model	2OGI
Resolution (Å) ^a^	1.91 (1.978–1.91)
No. reflections	35,247 (3499)
Rwork (Rfree) (%)	21.66/25.21 (28.44/33.72)
No. atoms	3433
Protein	3117
Ligands	54
Solvent	262
B-factors (Å2)	36.31
Protein	35.87
Ligands	31.85
Solvent	42.50
R.m.s. deviations	
Bond lengths (Å)	0.014
Bond angles (º)	1.68
% favored (allowed) in Ramachandran plot	98.71 (1.29)

^a^ Statistics for the highest-resolution shell are shown in parentheses.

**Table 2:** The effect of di-ion addition on the activity of *Sp-yqeK*.

Di-ions	(µmol/min/mg ± SD)
EDTA	0.0675 ± 0.0345
Zn^2+^	1.1418 ± 0.0693
Mg^2+^	1.3499 ± 0.0773
Ca^2+^	1.1987 ± 0.0577
Mn^2+^	1.3176 ± 0.0298
Ni^2+^	1.0316 ± 0.0194

**Table 3:** The effect of mutations at key sites of *Sp-yqeK* protein on its hydrolytic activity.

No	(µmol/min/mg ± SD)
WT	1.2165 ± 0.0886
H29A	0.0330 ± 0.0066
K62A	0.0505 ± 0.0043
H91A	0.0161 ± 0.0005
H116A	0.0421 ± 0.0024
N135A	0.1789 ± 0.0082
L172A	0.8819 ± 0.0390
F180A	0.6060 ± 0.3536
T183A	0.7765 ± 0.0075

**Table 4:** Comparison of kinetic data between WT-Sp-yqeK protein and various active mutant Sp-yqeK proteins.

Mutation	Km (µM ± SD)
WT	216.38842 ± 21.65011
L172A	1008.58396 ± 346.4237
F180A	1986.78696 ± 651.3617
T183A	1023.7566 ± 328.12593

## Data Availability

Protein Data Bank: The structure factor and coordinate for the complex of *Sp-yqeK*, ADP and Mg^2+^ have been deposited with accession code 8WMY (The PDB DOI for the deposition is 10.2210/pdb8wmy/pdb). The datasets and materials used and/or analyzed during the current study are available from the corresponding author upon reasonable request.

## Supplementary Materials

The following supporting information can be downloaded at: https://www.mdpi.com/article/10.3390/microorganisms13020230/s1, Figure S1: The SDS-PAGE of Sp-YqeK protein; Figure S2: Sequence alignment of YqeK across other species; Figure S3: Crystal structures of *Sp-yqeK*; Figure S4: Structural model depicting the Ap4A molecule in the binding pocket. Residues interacting with Ap4A are highlighted in purple; Table S1: List of protein-ligand interactions.
