# Supplementary material for: Investigation of the Structure and Functional Activity of the YqeK Protein in Streptococcus pyogenes with High Efficiency in Hydrolyzing Ap4A
Source: Microorganisms. 2025 Jan 22;13(2):230. doi: 10.3390/microorganisms13020230 (PMC11857287; doi:10.3390/microorganisms13020230)
Supplement: Supplementary file 1 [file microorganisms-13-00230-s001.zip › microorganisms-3422177-supplementary.pdf]

Supplementary Materials for

Investigation of the structure and functional activity of the YqeK protein in *Streptococcus pyogenes* with high efficiency in hydrolyzing Ap4A

Kai Yang[1, #], Suhua Hu[1, #], Yao Yao[1], Kaijie Li[1], Zunbao Wang[1], Xinyu Wang[1], Dan Ma[1], Mingfang Bi[1, *] and Xiaobing Mo[1, *]

*: To whom correspondence should be addressed to Mingfang Bi (bi_mingfang@gibh.ac.cn) and Xiaobing Mo (Email: mox@jlu.edu.cn).

This PDF file includes:

Supplementary Figure and Table Legends

Figure S1 to S5

Table S1

Supplementary Figures caption

Figure S1. The SDS-PAGE of *Sp*-YqeK protein.

Figure S2. Sequence alignment of YqeK across other species.

The detailed multiple sequence alignment of the YqeK from several strains is shown. The red background represents highly conserved sequences, red text indicates generally conserved sequences, and black text signifies non-conserved sequences. The secondary structure is shown above the aligned sequences, α-helices are displayed as helices.

Figure S3. Crystal structures of *Sp*-YqeK.

(**a**) Crystal structure of *Halalkalibacterium halodurans* YqeK complexed with FE and ADP-like ligand (PDB: 2O08). YqeK, FE, and the ADP-like ligand are shown in red, green, and yellow, respectively. (**b**) Crystal structure of *Streptococcus agalactiae* YqeK complexed with GDP and FE (PDB: 2OGI). YqeK, FE, and GDP are shown in deep blue, green, and yellow, respectively. (**c**) Crystal structure of *Clostridium acetobutylicum* YqeK complexed with GDP and PO_4^{3+} (PDB: 3CCG). YqeK, FE, and PO4 are shown in purple, green, and yellow, respectively. (**d**) Crystal structure of *Streptococcus pyogenes* YqeK complexed with Mg^{2+} and ADP (PDB: 8WMY). YqeK, MG, and ADP are shown in gray, brown, and yellow, respectively.

Figure S4. Structural model depicting the Ap4A molecule in the binding pocket. Residues interacting with Ap4A are highlighted in purple.

Table S1.List of protein-ligand interactions

Hydrogen bonds

	<----- A T O M 1 ----->						<----- A T O M 2 ----->					
	Atom no.	Atom name	Res name	Res no.	Chain		Atom no.	Atom name	Res name	Res no.	Chain	Distance
1.	248	NE2	HIS	29	A	-->	3131	O3B	ADP	201	A	2.71
2.	248	NE2	HIS	29	A	<--	3131	O3B	ADP	201	A	2.71
3.	451	NE2	HIS	58	A	-->	3129	O1B	ADP	201	A	3.12
4.	458	OD1	ASP	59	A	<--	3130	O2B	ADP	201	A	3.23
5.	459	OD2	ASP	59	A	<--	3131	O3B	ADP	201	A	2.97
6.	485	NZ	LYS	62	A	-->	3130	O2B	ADP	201	A	3.08
7.	702	ND2	ASN	88	A	-->	3153	N3	ADP	201	A	3.29
8.	733	NE2	HIS	91	A	-->	3134	O2A	ADP	201	A	2.92
9.	940	NE2	HIS	117	A	-->	3129	O1B	ADP	201	A	3.02
10.	940	NE2	HIS	117	A	-->	3134	O2A	ADP	201	A	3.02
11.	940	NE2	HIS	117	A	<--	3134	O2A	ADP	201	A	3.02
12.	946	OG1	THR	118	A	-->	3134	O2A	ADP	201	A	2.73
13.	946	OG1	THR	118	A	<--	3134	O2A	ADP	201	A	2.73
14.	1071	OD1	ASP	135	A	<--	3131	O3B	ADP	201	A	3.06
15.	1419	N	PHE	180	A	-->	3150	N6	ADP	201	A	2.93
16.	1451	OG1	THR	183	A	-->	3150	N6	ADP	201	A	2.80
17.	1451	OG1	THR	183	A	<--	3150	N6	ADP	201	A	2.80

Non-bonded contacts

	<----- A T O M 1 ----->						<----- A T O M 2 ----->					
	Atom no.	Atom name	Res name	Res no.	Chain		Atom no.	Atom name	Res name	Res no.	Chain	Distance
1.	246	CD2	HIS	29	A	---	3131	O3B	ADP	201	A	3.55
2.	247	CE1	HIS	29	A	---	3131	O3B	ADP	201	A	3.31
3.	248	NE2	HIS	29	A	---	3131	O3B	ADP	201	A	2.71
4.	449	CD2	HIS	58	A	---	3129	O1B	ADP	201	A	3.07
5.	451	NE2	HIS	58	A	---	3129	O1B	ADP	201	A	3.12
6.	457	CG	ASP	59	A	---	3128	PB	ADP	201	A	3.74
7.	457	CG	ASP	59	A	---	3129	O1B	ADP	201	A	3.36
8.	457	CG	ASP	59	A	---	3130	O2B	ADP	201	A	3.64
9.	458	OD1	ASP	59	A	---	3128	PB	ADP	201	A	3.52
10.	458	OD1	ASP	59	A	---	3129	O1B	ADP	201	A	2.97
11.	458	OD1	ASP	59	A	---	3130	O2B	ADP	201	A	3.23

12.	459	OD2	ASP	59	A	---	3128	PB	ADP	201	A	3.15
13.	459	OD2	ASP	59	A	---	3129	O1B	ADP	201	A	3.02
14.	459	OD2	ASP	59	A	---	3130	O2B	ADP	201	A	3.39
15.	459	OD2	ASP	59	A	---	3131	O3B	ADP	201	A	2.97
16.	483	CD	LYS	62	A	---	3130	O2B	ADP	201	A	3.77
17.	484	CE	LYS	62	A	---	3130	O2B	ADP	201	A	3.76
18.	485	NZ	LYS	62	A	---	3130	O2B	ADP	201	A	3.08
19.	485	NZ	LYS	62	A	---	3137	C5'	ADP	201	A	3.49
20.	699	CB	ASN	88	A	---	3139	O4'	ADP	201	A	3.24
21.	699	CB	ASN	88	A	---	3144	C1'	ADP	201	A	3.87
22.	699	CB	ASN	88	A	---	3153	N3	ADP	201	A	3.71
23.	700	CG	ASN	88	A	---	3138	C4'	ADP	201	A	3.54
24.	700	CG	ASN	88	A	---	3139	O4'	ADP	201	A	3.08
25.	700	CG	ASN	88	A	---	3144	C1'	ADP	201	A	3.62
26.	701	OD1	ASN	88	A	---	3138	C4'	ADP	201	A	3.38
27.	701	OD1	ASN	88	A	---	3139	O4'	ADP	201	A	3.39
28.	702	ND2	ASN	88	A	---	3139	O4'	ADP	201	A	3.44
29.	702	ND2	ASN	88	A	---	3144	C1'	ADP	201	A	3.44
30.	702	ND2	ASN	88	A	---	3153	N3	ADP	201	A	3.29
31.	732	CE1	HIS	91	A	---	3130	O2B	ADP	201	A	3.90
32.	732	CE1	HIS	91	A	---	3134	O2A	ADP	201	A	3.05
33.	733	NE2	HIS	91	A	---	3130	O2B	ADP	201	A	3.35
34.	733	NE2	HIS	91	A	---	3134	O2A	ADP	201	A	2.92
35.	938	CD2	HIS	117	A	---	3129	O1B	ADP	201	A	3.17
36.	938	CD2	HIS	117	A	---	3134	O2A	ADP	201	A	3.42
37.	940	NE2	HIS	117	A	---	3129	O1B	ADP	201	A	3.02
38.	940	NE2	HIS	117	A	---	3134	O2A	ADP	201	A	3.02
39.	942	CA	THR	118	A	---	3133	O1A	ADP	201	A	3.79
40.	945	CB	THR	118	A	---	3134	O2A	ADP	201	A	3.62
41.	946	OG1	THR	118	A	---	3132	PA	ADP	201	A	3.78
42.	946	OG1	THR	118	A	---	3134	O2A	ADP	201	A	2.73
43.	947	CG2	THR	118	A	---	3132	PA	ADP	201	A	3.70
44.	947	CG2	THR	118	A	---	3133	O1A	ADP	201	A	3.61
45.	947	CG2	THR	118	A	---	3134	O2A	ADP	201	A	3.51
46.	947	CG2	THR	118	A	---	3136	O5'	ADP	201	A	3.52
47.	947	CG2	THR	118	A	---	3139	O4'	ADP	201	A	3.60
48.	947	CG2	THR	118	A	---	3146	C8	ADP	201	A	3.51
49.	1070	CG	ASP	135	A	---	3128	PB	ADP	201	A	3.87
50.	1070	CG	ASP	135	A	---	3129	O1B	ADP	201	A	3.16
51.	1070	CG	ASP	135	A	---	3131	O3B	ADP	201	A	3.80
52.	1071	OD1	ASP	135	A	---	3128	PB	ADP	201	A	3.53
53.	1071	OD1	ASP	135	A	---	3129	O1B	ADP	201	A	2.98
54.	1071	OD1	ASP	135	A	---	3131	O3B	ADP	201	A	3.06
55.	1072	OD2	ASP	135	A	---	3128	PB	ADP	201	A	3.40

56.	1072	OD2	ASP	135	A	---	3129	O1B	ADP	201	A	2.76
57.	1072	OD2	ASP	135	A	---	3131	O3B	ADP	201	A	3.75
58.	1072	OD2	ASP	135	A	---	3132	PA	ADP	201	A	3.64
59.	1072	OD2	ASP	135	A	---	3133	O1A	ADP	201	A	3.11
60.	1072	OD2	ASP	135	A	---	3135	O3A	ADP	201	A	3.33
61.	1337	CG2	THR	168	A	---	3146	C8	ADP	201	A	3.73
62.	1337	CG2	THR	168	A	---	3147	N7	ADP	201	A	3.82
63.	1368	CD1	LEU	172	A	---	3148	C5	ADP	201	A	3.55
64.	1368	CD1	LEU	172	A	---	3149	C6	ADP	201	A	3.60
65.	1369	CD2	LEU	172	A	---	3152	C2	ADP	201	A	3.64
66.	1369	CD2	LEU	172	A	---	3153	N3	ADP	201	A	3.54
67.	1369	CD2	LEU	172	A	---	3154	C4	ADP	201	A	3.90
68.	1407	O	PRO	178	A	---	3151	N1	ADP	201	A	2.91
69.	1407	O	PRO	178	A	---	3152	C2	ADP	201	A	3.28
70.	1412	CA	ILE	179	A	---	3150	N6	ADP	201	A	3.78
71.	1413	C	ILE	179	A	---	3150	N6	ADP	201	A	3.81
72.	1419	N	PHE	180	A	---	3149	C6	ADP	201	A	3.71
73.	1419	N	PHE	180	A	---	3150	N6	ADP	201	A	2.93
74.	1419	N	PHE	180	A	---	3151	N1	ADP	201	A	3.73
75.	1420	CA	PHE	180	A	---	3150	N6	ADP	201	A	3.75
76.	1423	CB	PHE	180	A	---	3150	N6	ADP	201	A	3.46
77.	1450	CB	THR	183	A	---	3150	N6	ADP	201	A	3.44
78.	1451	OG1	THR	183	A	---	3150	N6	ADP	201	A	2.80
79.	1452	CG2	THR	183	A	---	3150	N6	ADP	201	A	3.83

Number of hydrogen bonds: 17

Number of non-bonded contacts: 79

Figure S1

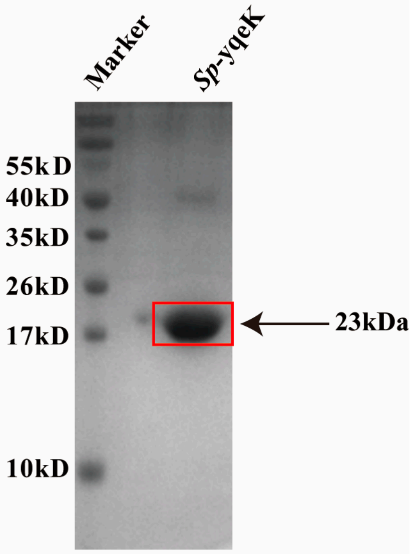

SDS-PAGE of *Sp*-YqeK

Figure S2

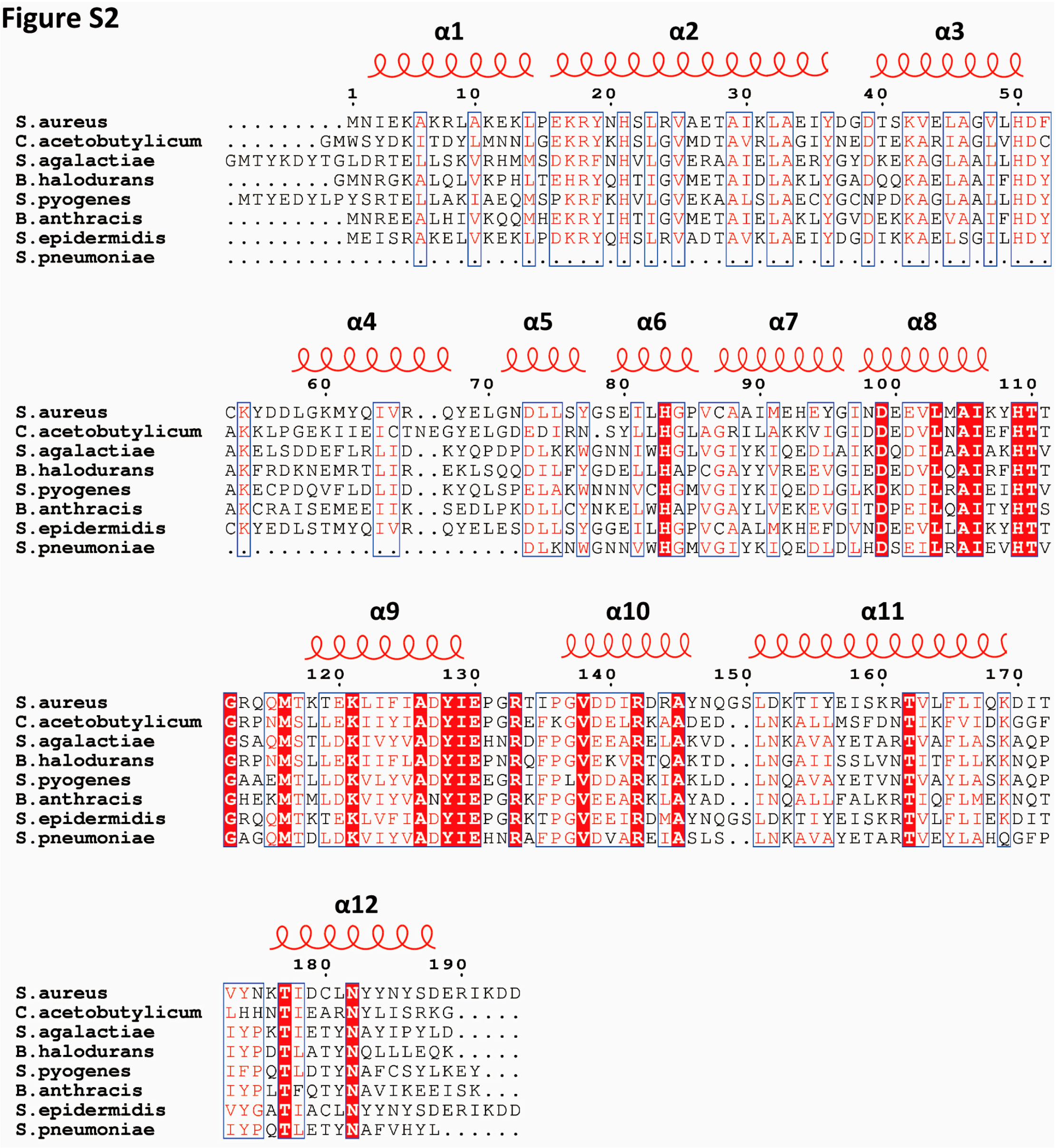

α1 α2 α3
1 10 20 30 40 50
S.aureus .........MNIEKAKRLAKEKLPEKRYNHSLRVAETAIKLAEIYDGDTSKVELAGVLHDF
C.acetobutylicum .......GMWSYDKITDYLMNNLGEKRYKHSLGVMDTAVRLAGIYNEDTEKARIAGLVHDC
S.agalactiae GMTYKDYTGLDRTELLSKVRHMMSDKRFNHVLGVERAAIELAERYGYDKEKAGLAALLHDY
B.halodurans ........GMNRGKALQLVKPHLTEHRYQHTIGVMETAIDLAKLYGADQQKAELAAIFHDY
S.pyogenes .MTYEDYLPYSRTELLAKIAEQMSPKRFKHVLGVEKAALSLAECYGCNPDKAGLAALLHDY
B.anthracis .........MNREEALHIVKQQMHEKRYIHTIGVMETAIELAKLYGVDEKKAEVAAIFHDY
S.epidermidis .........MEISRAKELVKEKLPDKRYQHSLRVADTAVKLAEIYDGDIKKAELSGILHDY
S.pneumoniae ....
α4 α5 α6 α7 α8
60 70 80 90 100 110
S.aureus CKYDDLGKMYQIVR..QYELGNDLLSYGSEILHGPVCAAIMEHEYGINDEEVLMAIKYHTT
C.acetobutylicum AKKLPGEKIIEICTNEGYELGDEDIRN.SYLLHGLAGRILAKKVIGIDDEDVLNAIEFHTT
S.agalactiae AKELSDDEFLRLID..KYQPDPDLKKWGNNIWHGLVGIYKIQEDLAIKDQDILAAIAKHTV
B.halodurans AKFRDKNEMRTLIR..EKLSQQDILFYGDELLHAPCGAYYVREEVGIEDEDVLQAIRFHTT
S.pyogenes AKECPDQVFLDLID..KYQLSPELAKWNNNVCHGMVGIYKIQEDLGLKDKDILRAIEIHTV
B.anthracis AKCRAISEMEEIIK..SEDLPKDLLCYNKELWHAPVGAYLVEKEVGITDPEILQAITYHTS
S.epidermidis CKYEDLSTMYQIVR..QYELESDLLSYGGEILHGPVCAALMKHEFDVNDEEVLLAIKYHTT
S.pneumoniae ......................DLKNWGNNVWHGMVGIYKIQEDLDLHDSEILRAIEVHTV
α9 α10 α11
120 130 140 150 160 170
S.aureus GRQQMTKTEKLIFIADYIEPGRTIPGVDDIRDRAYNQGSLDKTIYEISKRTVLFLIQKDIT
C.acetobutylicum GRPNMSLLEKIIYIADYIEPGREFKGVDELRKAADED..LNKALLMSFDNTIKFVIDKGGF
S.agalactiae GSAQMSTLDKIVYVADYIEHNRDFPGVEEARELAKVD..LNKAVAYETARTVAFLASKAQP
B.halodurans GRPNMSLLEKIIFLADYIEPNRQFPGVEKVRTQAKTD..LNGAIISSLVNTITFLLKKNQP
S.pyogenes GAAEMTLLDKVLYVADYIEEGRIFPLVDDARKIAKLD..LNQAVAYETVNTVAYLASKAQP
B.anthracis GHEKMTMLDKVIYVANYIEPGRKFPGVEEARKLAYAD..INQALLFALKRTIQFLMEKNQT
S.epidermidis GRQQMTKTEKLVFIADYIEPGRKTPGVEEIRDMAYNQGSLDKTIYEISKRTVLFLIEKDIT
S.pneumoniae GAGQMTDLDKVIYVADYIEHNRAFPGVDVAREIASLS..LNKAVAYETARTVEYLAHQGFP
α12
180 190
S.aureus VYNKTIDCLNYYNYSDERIKDD
C.acetobutylicum LHHNTIEARNYLISRKG.....
S.agalactiae IYPKTIETYNAYIPYLD.....
B.halodurans IYPDTLATYNQLLLEQK.....
S.pyogenes IFPQTLDTYNAFCSYLKEY...
B.anthracis IYPLTFQTYNAVIKEEISK...
S.epidermidis VYGATIACLNYYNYSDERIKDD
S.pneumoniae IYPQTLETYNAFVHYL......

Figure S3

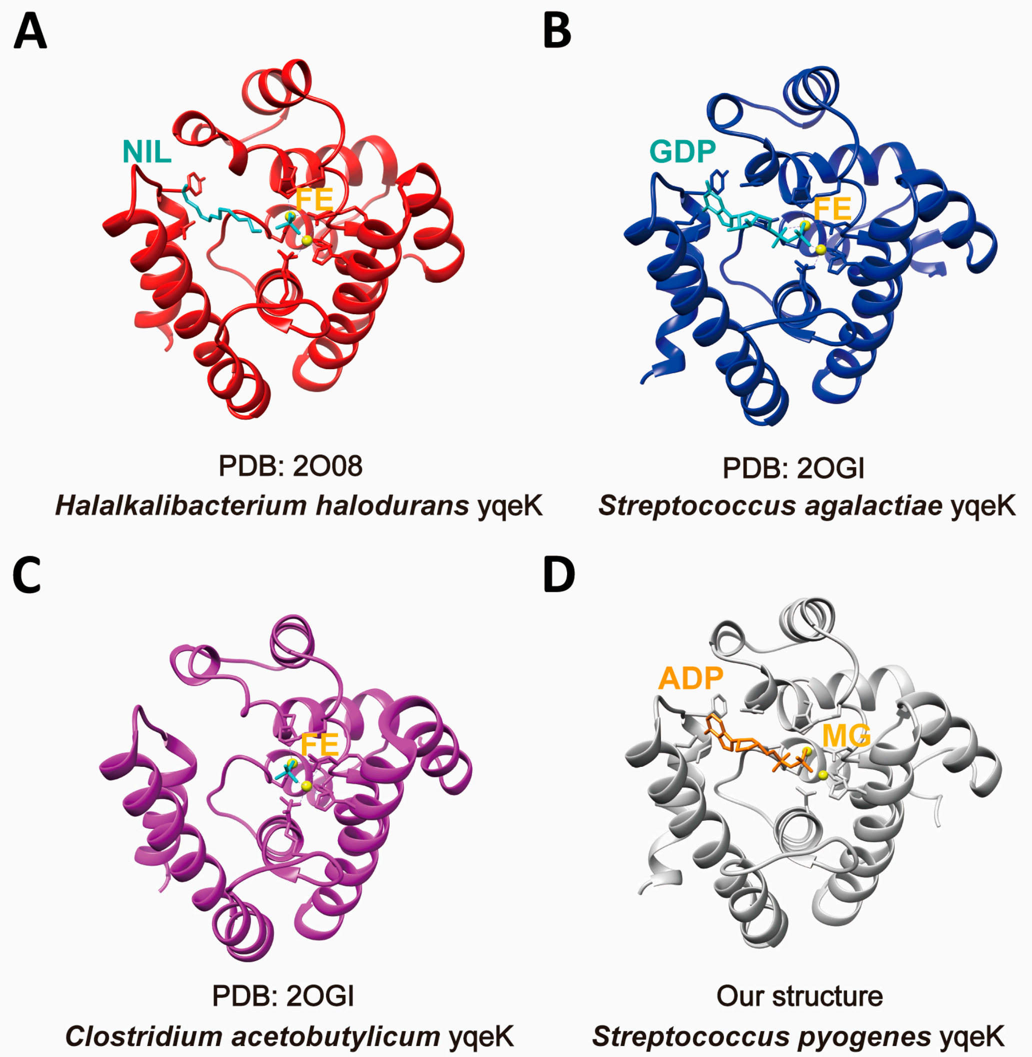
A
NIL
FE
PDB: 2O08
Halalkalibacterium halodurans yqeK
B
GDP
FE
PDB: 2OGI
Streptococcus agalactiae yqeK
C
FE
PDB: 2OGI
Clostridium acetobutylicum yqeK
D
ADP
MG
Our structure
Streptococcus pyogenes yqeK

Figure S4

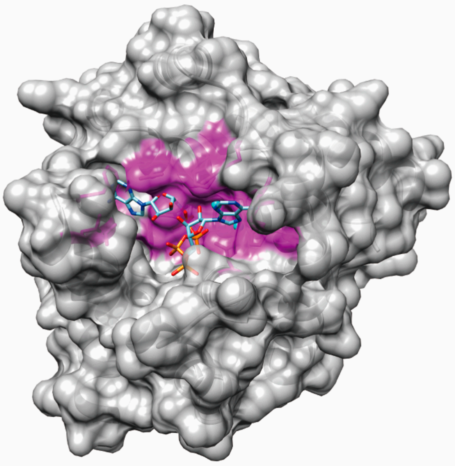

Structural model depicting the Ap4A molecule in the binding pocket
